# Supplementary figures and images for: Impact of nanodisc lipid composition on cell-free expression of proton-coupled folate transporter
Source: PLoS One. 2021 Nov 18;16(11):e0253184. doi: 10.1371/journal.pone.0253184 (PMC8601550; doi:10.1371/journal.pone.0253184)

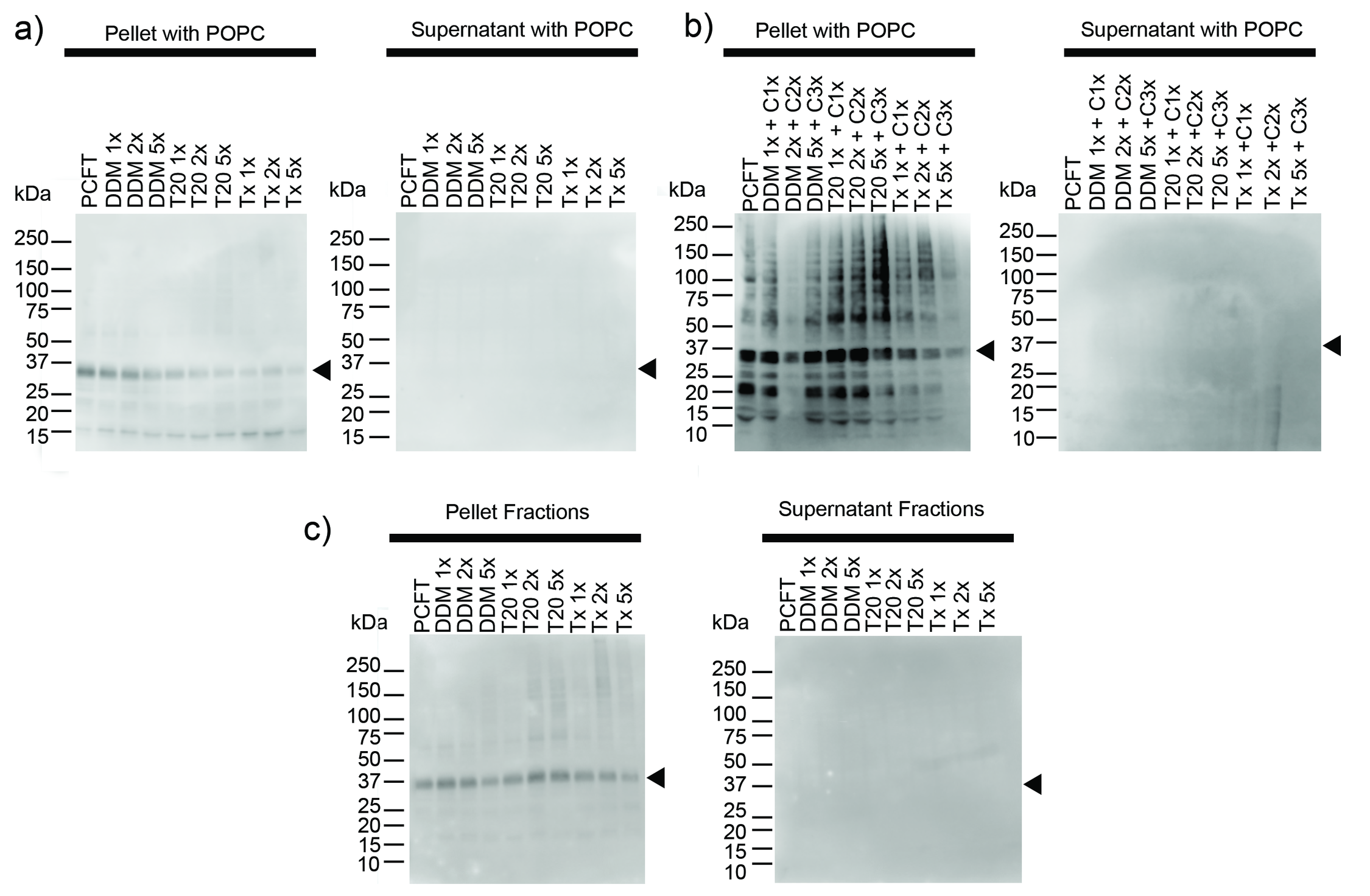

Supplement: S1 Fig — (TIF) [file pone.0253184.s001.tif]
