# Supplementary material for: Impact of nanodisc lipid composition on cell-free expression of proton-coupled folate transporter
Source: PLoS One. 2021 Nov 18;16(11):e0253184. doi: 10.1371/journal.pone.0253184 (PMC8601550; doi:10.1371/journal.pone.0253184)
Supplement: S1 File — (DOCX) [file pone.0253184.s002.docx]

Supporting Information

Cell-free expression of proton-coupled folate transporter
in the presence of nanodiscs

Hoa Quynh Do^1^, Carla M. Bassil^1,2^, Elizabeth I. Andersen^1^, Michaela Jansen^1^*

^1^Department of Cell Physiology and Molecular Biophysics and Center for Membrane Protein Research, School of Medicine, Texas Tech University Health Sciences Center, Lubbock, Texas

^2^The Clark Scholar Program, Texas Tech University, Lubbock, TX 79409, USA

*Corresponding author.

Email: [michaela.jansen@ttuhsc.edu](mailto:michaela.jansen@ttuhsc.edu) (MJ)


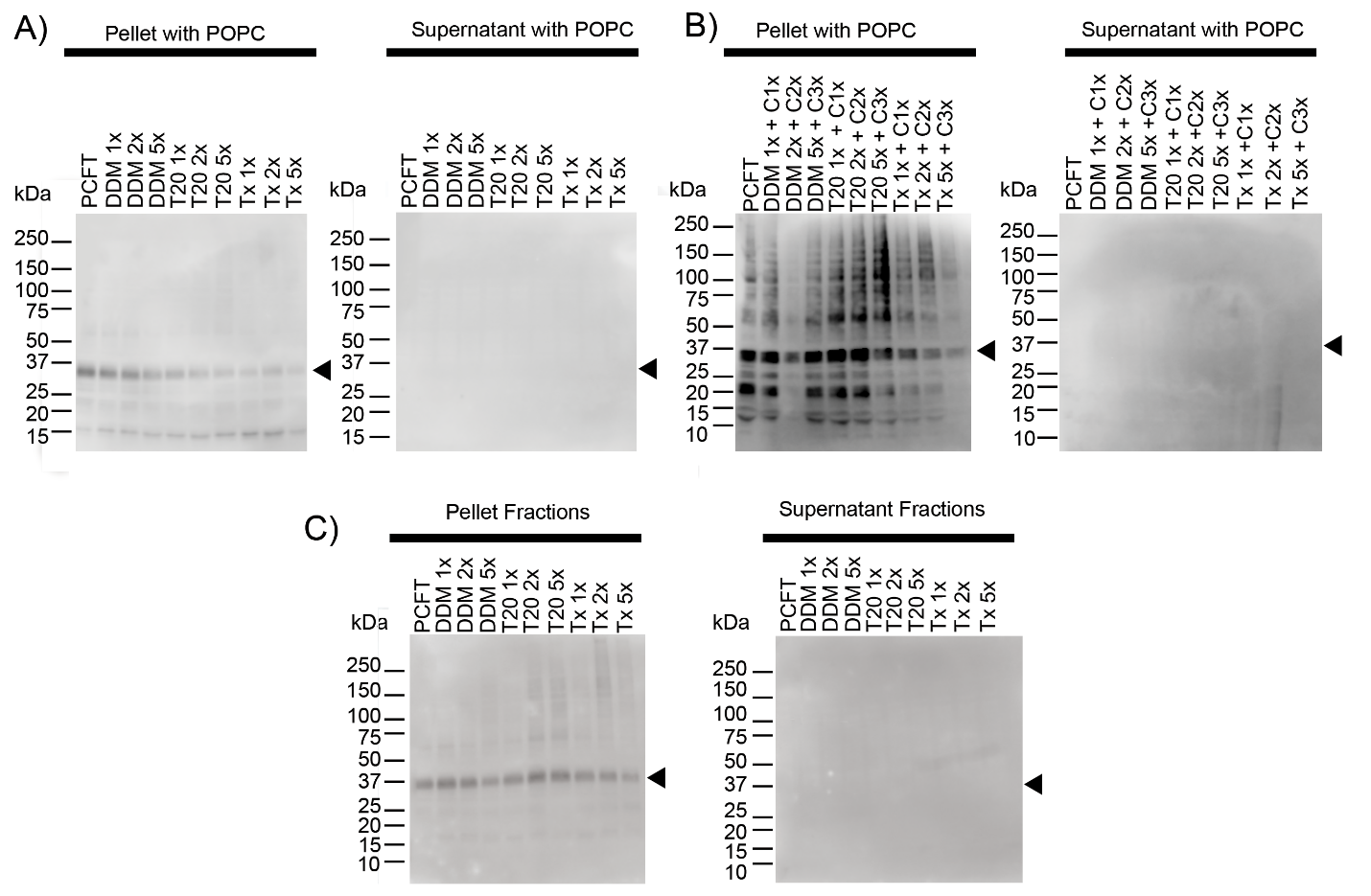


Figure S1. Cell-free expression of PCFT in the presence of POPC lipid with and without detergent or detergent alone. A) PCFT was expressed in the presence of both POPC lipid and detergent. B) PCFT was expressed in the presence of both POPC lipid and a detergent, and cholic acid was added after the expression. C) PCFT was expressed in the presence of DDM, Tween 20 or Triton X-100. The arrows indicate the position of monomeric PCFT (~37 kDa). POPC concentrations were 1.05 mM, 0.53 mM, and 0.21 mM in combination with 5 times, 2 times, and 1 time of detergent critical micelle concentration, respectively. Tween 20 (Tw20), Triton X-100 (Tx) or cholic acid (labeled as C in the blot images) was used at one time (1x), two times (2x), three times (3x), or five times (5x) of the detergents’ or cholic acid’s critical micelle concentration.
