# Supplementary material for: Impact of nanodisc lipid composition on cell-free expression of proton-coupled folate transporter
Source: PLoS One. 2021 Nov 18;16(11):e0253184. doi: 10.1371/journal.pone.0253184 (PMC8601550; doi:10.1371/journal.pone.0253184)

Supporting Information

Cell-free expression of proton-coupled folate transporter in the presence of nanodiscs

Hoa Quynh Do[1], Carla M. Bassil[1,2], Elizabeth I. Andersen[1], Michaela Jansen[1]*

[1]Department of Cell Physiology and Molecular Biophysics and Center for Membrane Protein Research, School of Medicine, Texas Tech University Health Sciences Center, Lubbock, Texas

[2]The Clark Scholar Program, Texas Tech University, Lubbock, TX 79409, USA

*Corresponding author.

Email: michaela.jansen@ttuhsc.edu (MJ)

This file contains original uncropped and unadjusted images

Original images used to build 3A

(2 images of protein ladders and PCFT immunoblots)

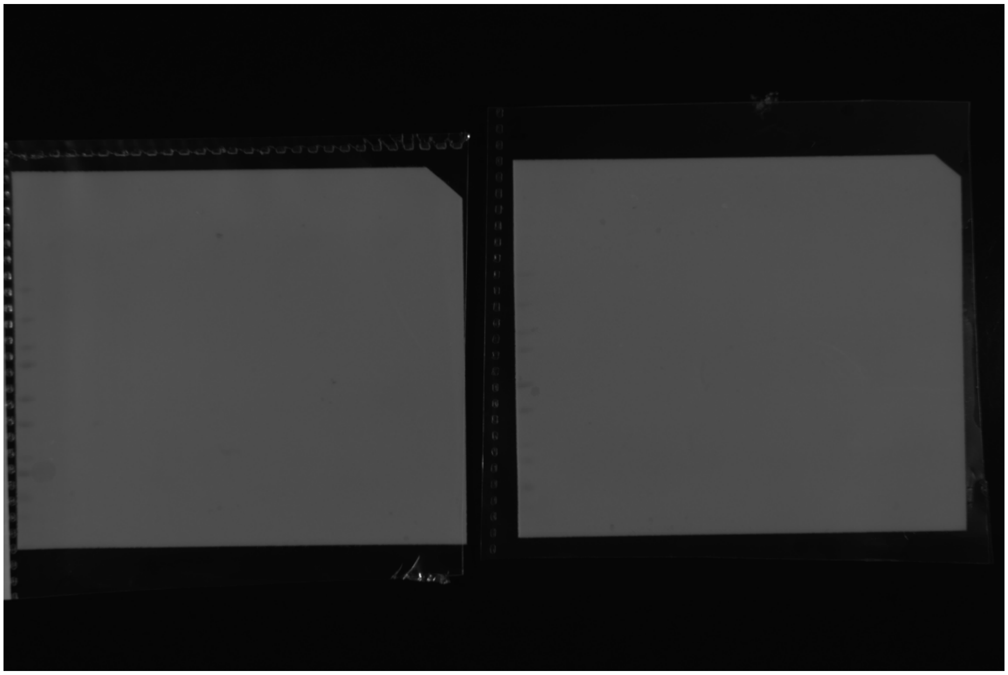

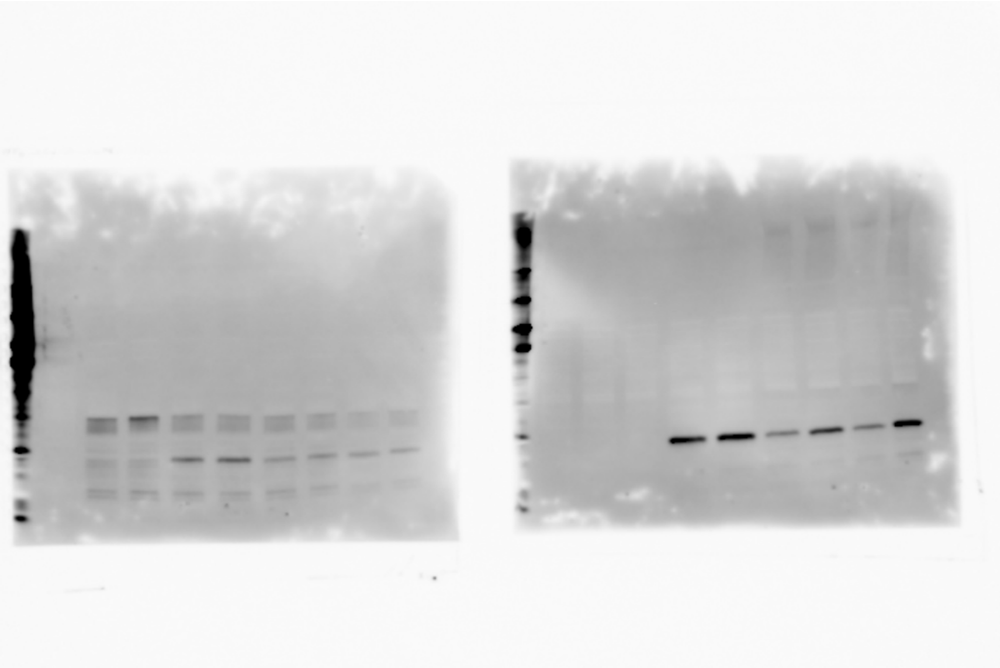

Original images used to build 3B

(2 images of protein ladders and PCFT immunoblots)

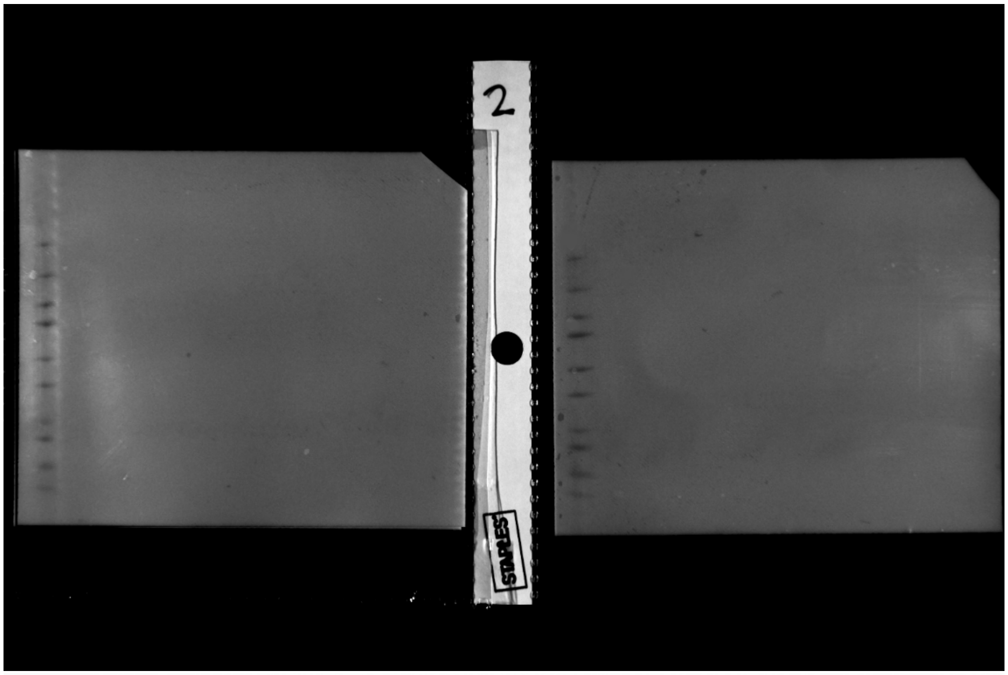

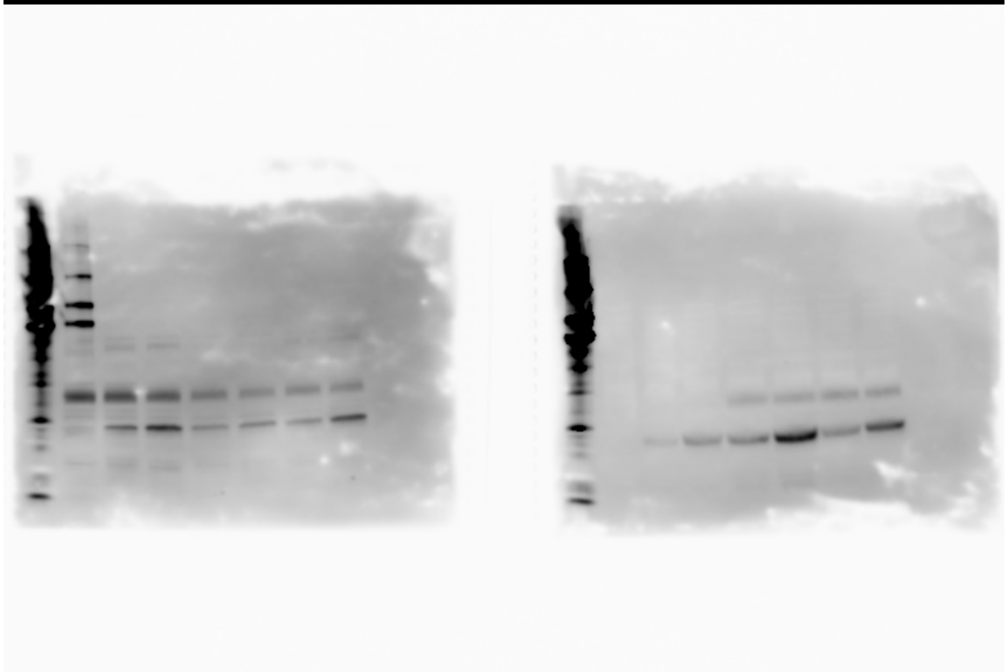

Original images used to build 4A

(2 images of protein ladders and PCFT immunoblots)

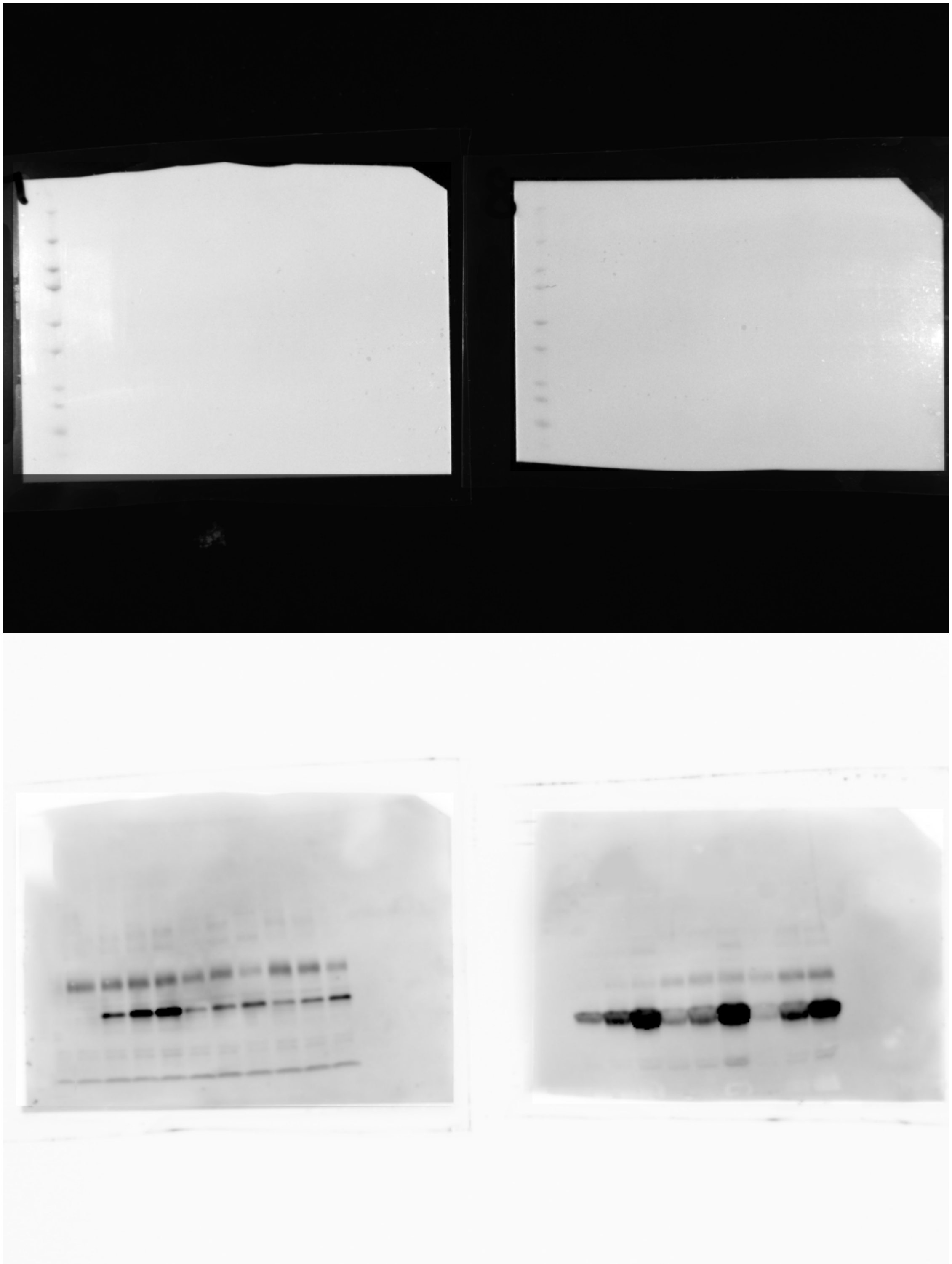

Original images used to build 4B

There are 3 separate data sets, each set has 2 images of protein ladders and PCFT immunoblots

Data set #1

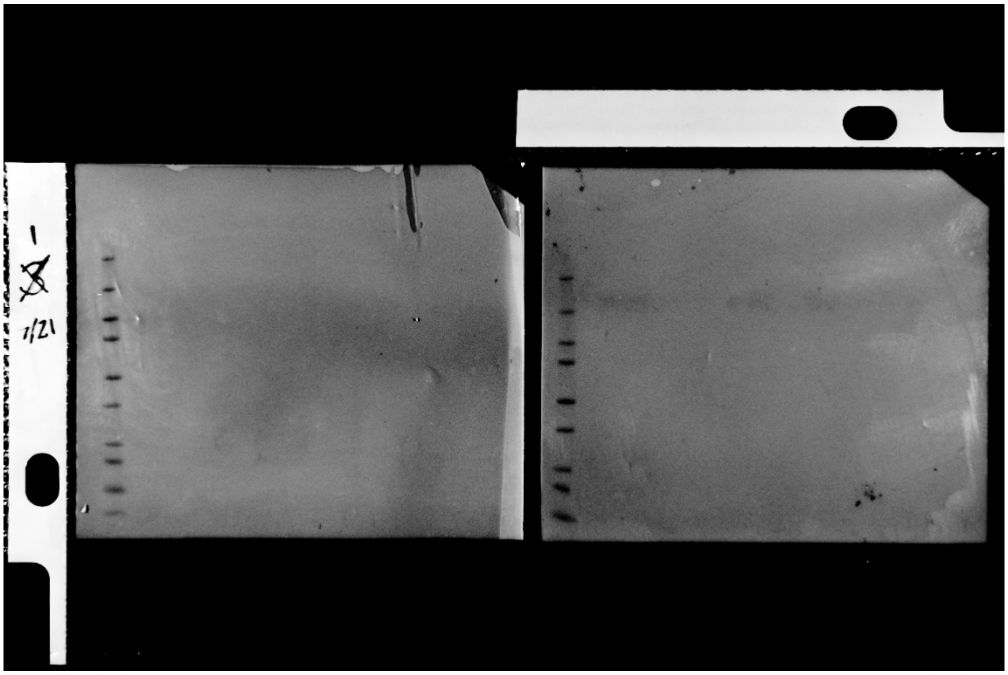

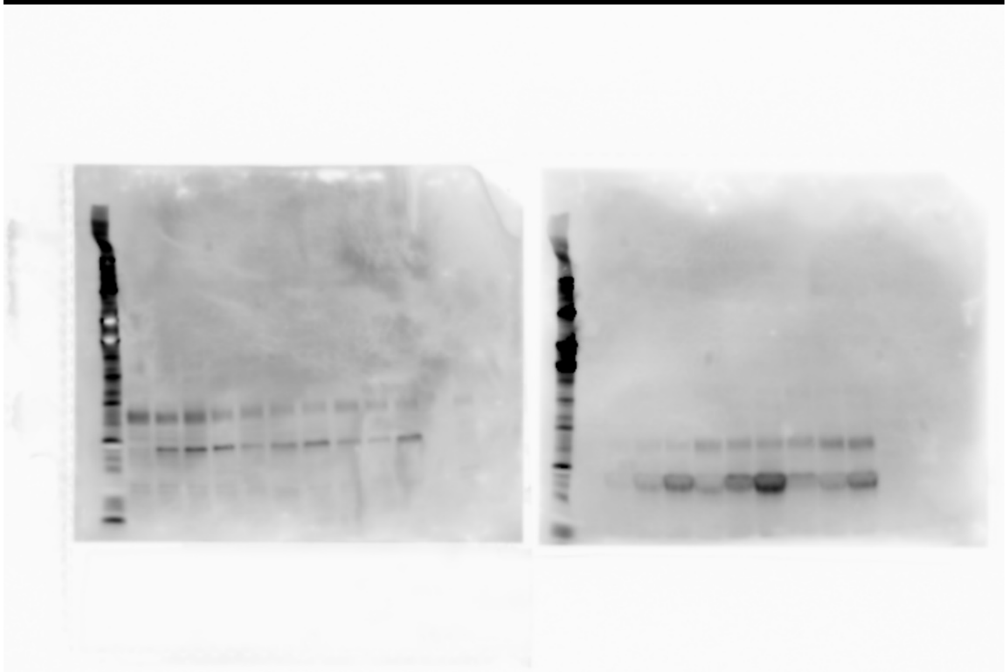

Original images used to build 4B

There are 3 separate data sets, each set has 2 images of protein ladders and PCFT immunoblots

Data set #2

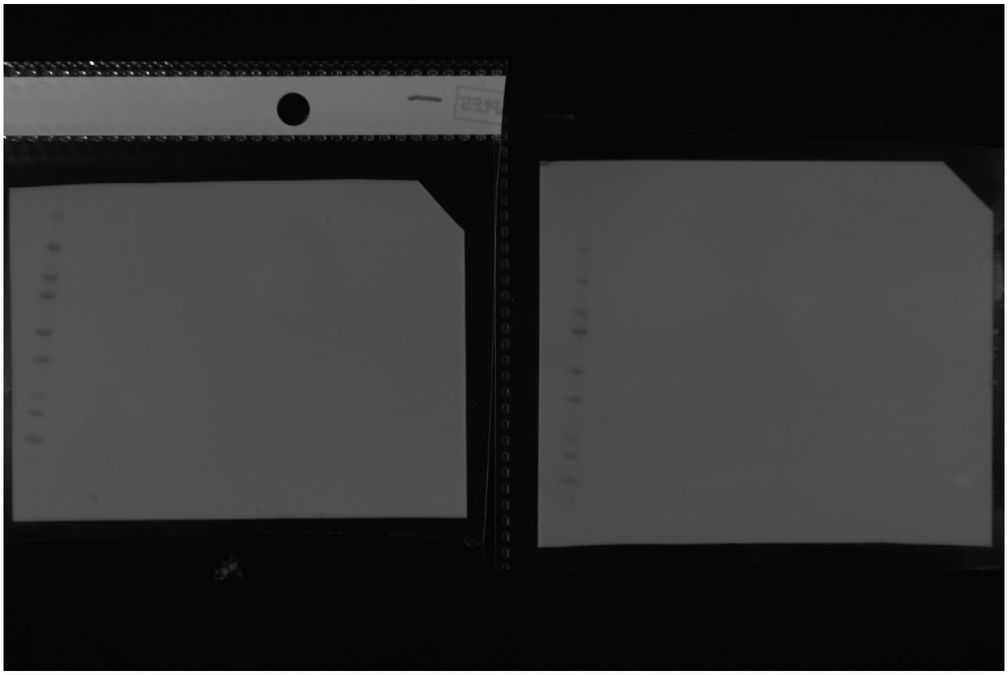

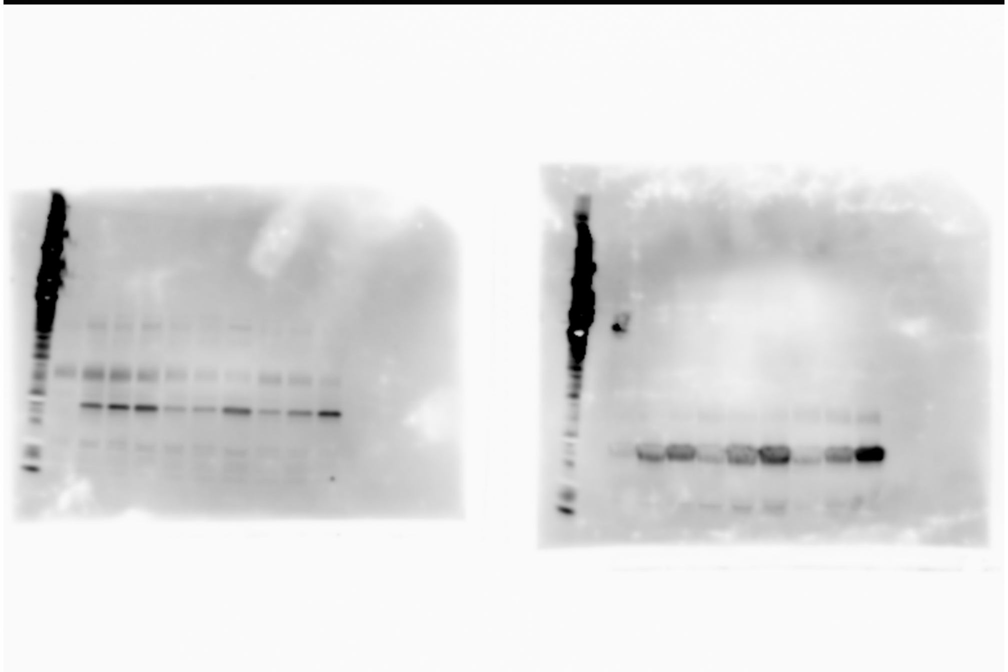

Original images used to build 4B

There are 3 separate data sets, each set has 2 images of protein ladders and PCFT immunoblots

Data set #3

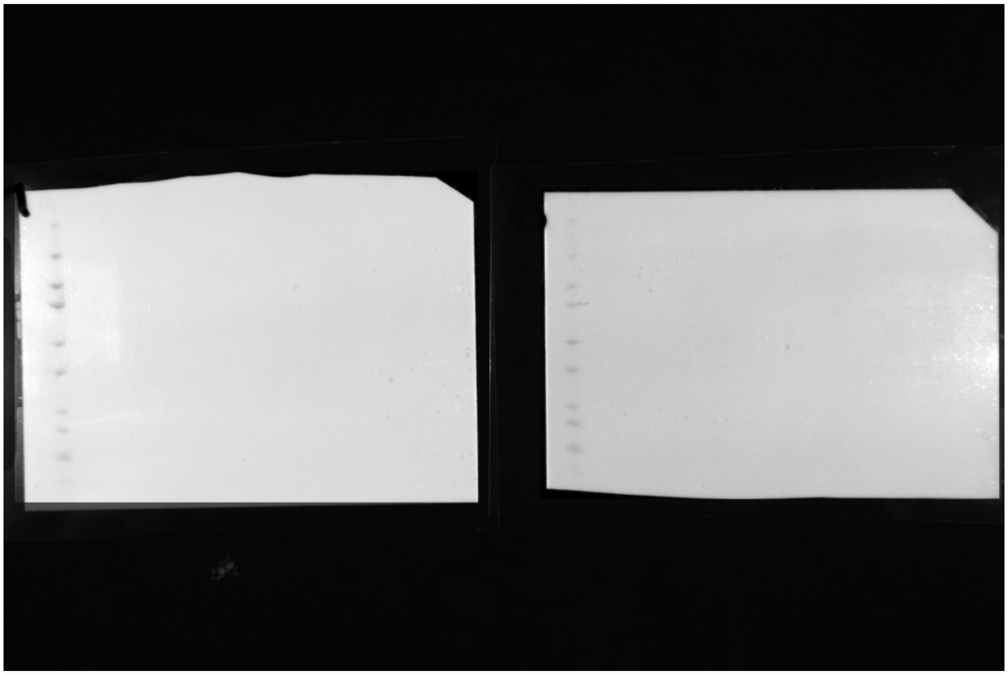

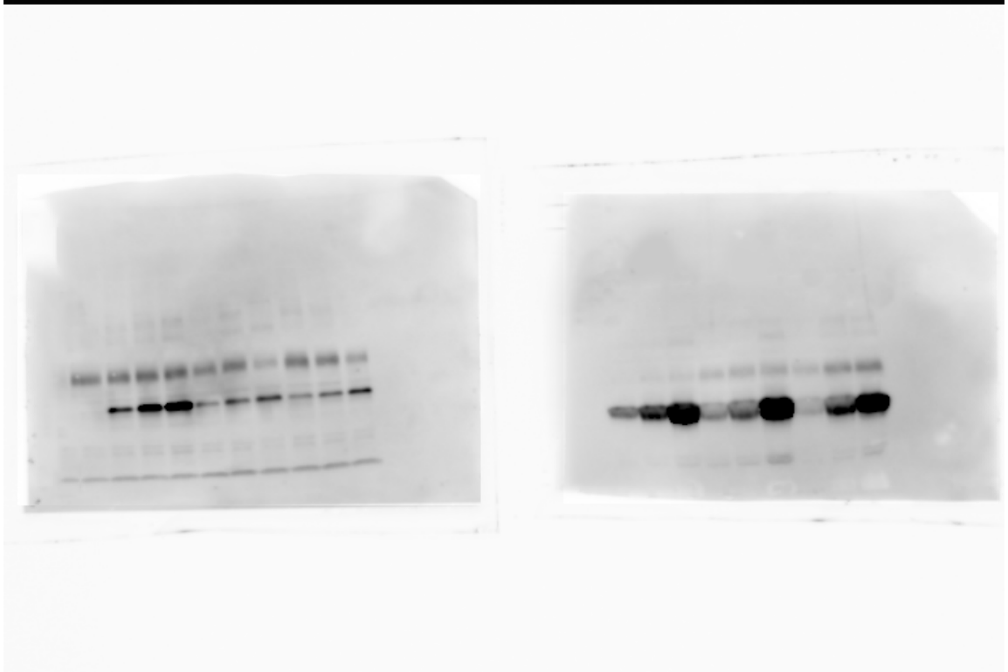

Original images used to build Figure S1-a

(2 images of protein ladders and PCFT immunoblots)

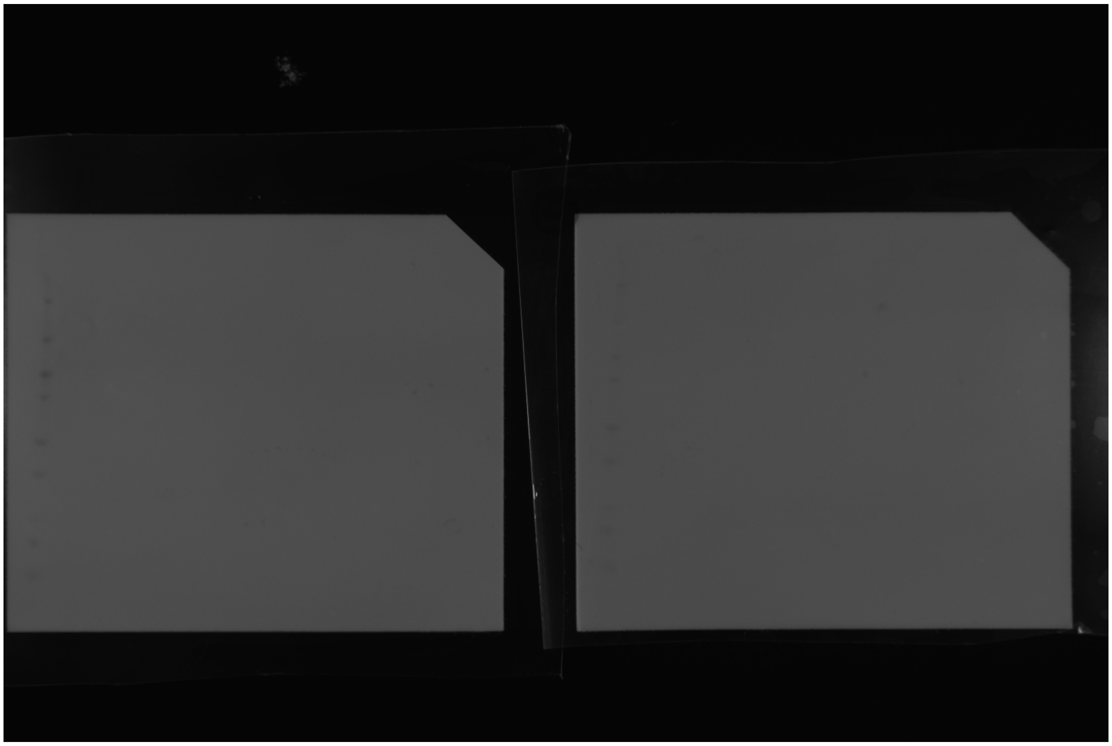

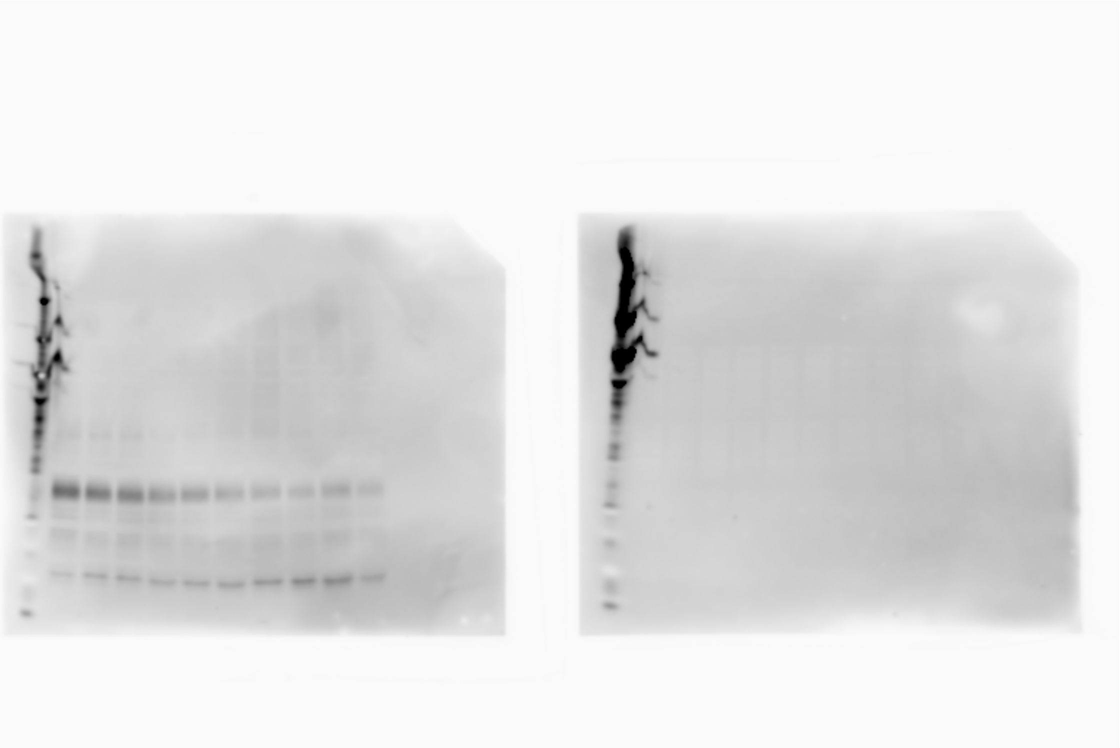

Original images used to build Figure S1-b

(2 images of protein ladders and PCFT immunoblots)

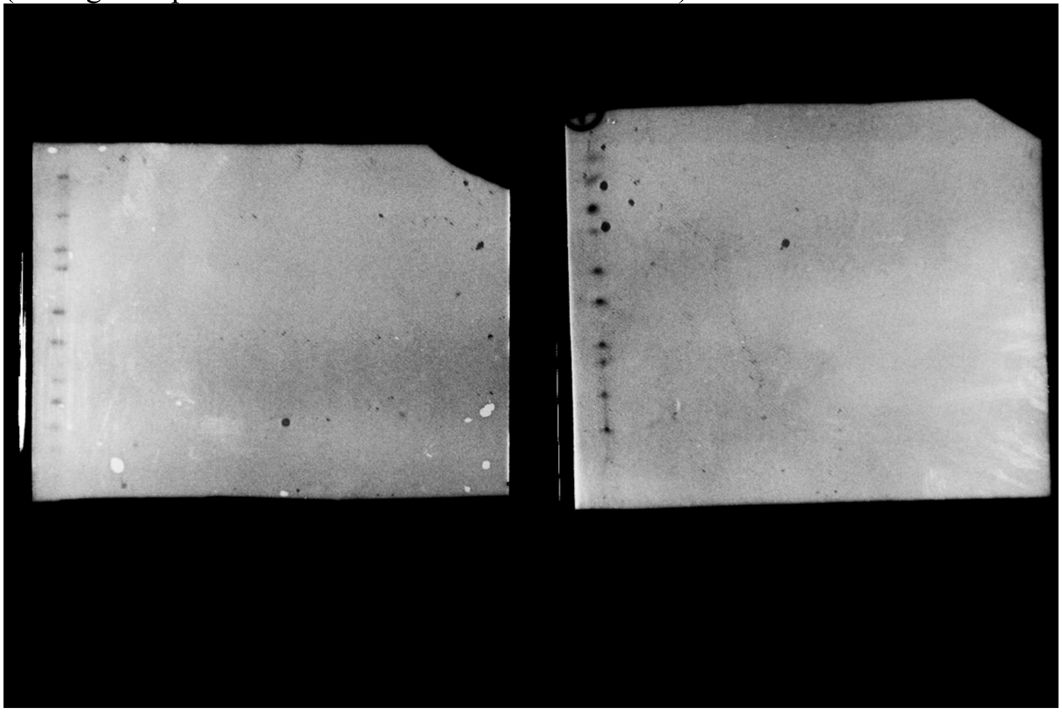

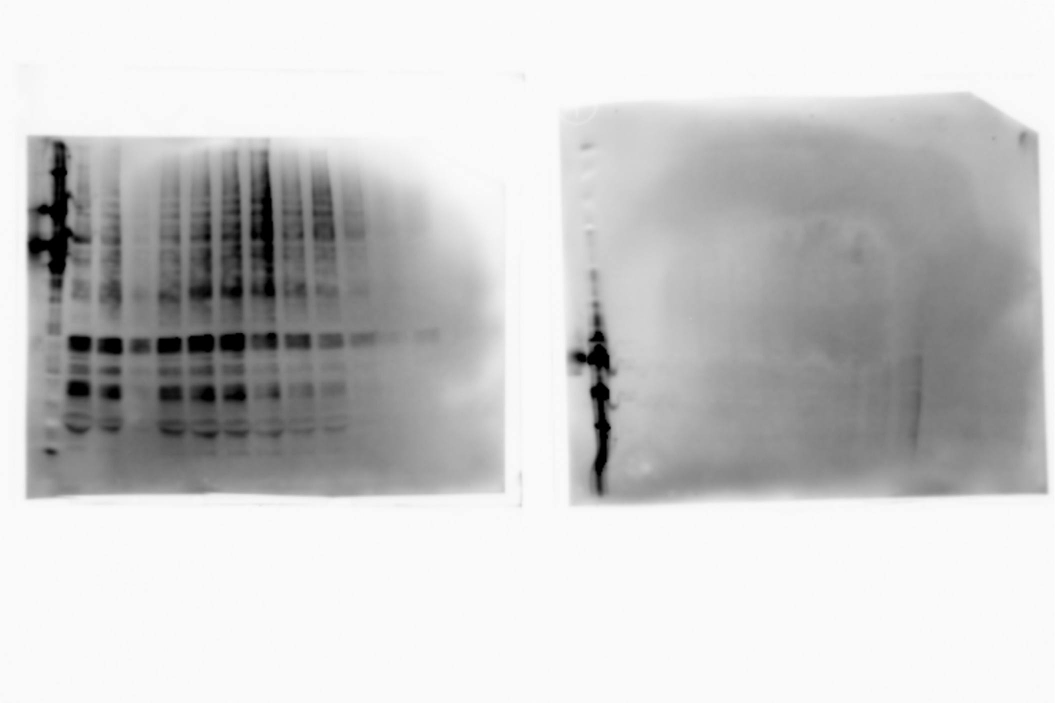

Original images used to build Figure S1-c

(2 images of protein ladders and PCFT immunoblots)

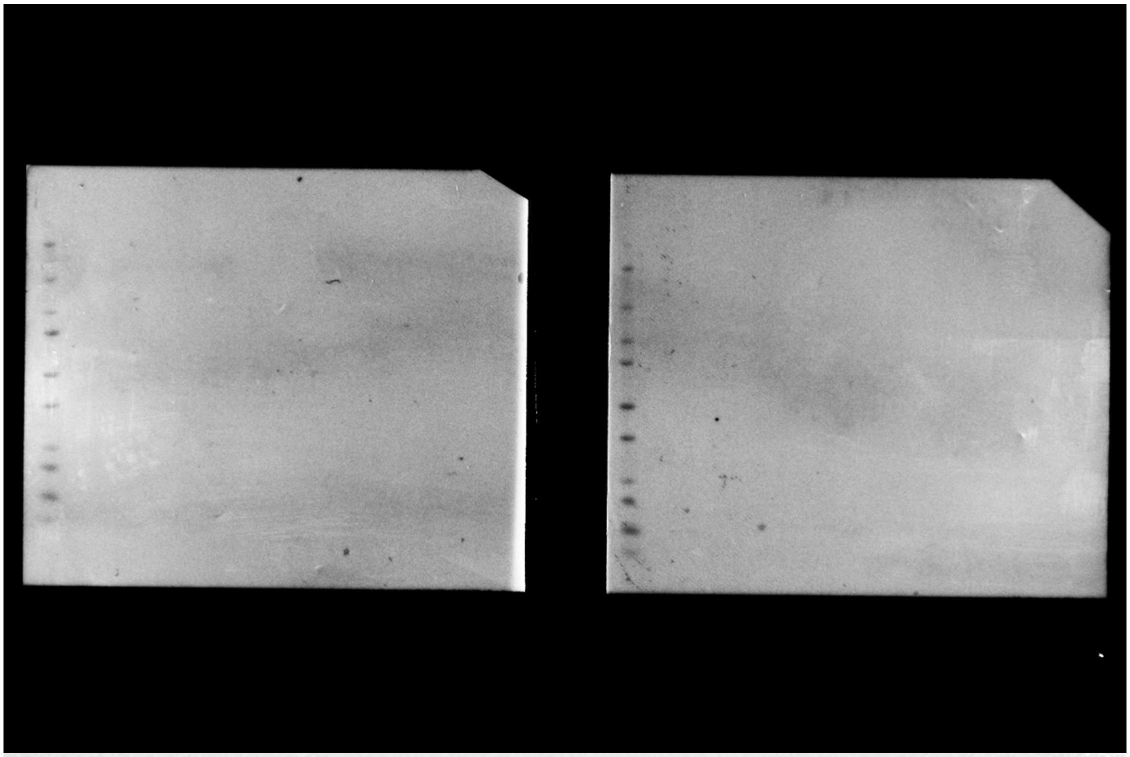

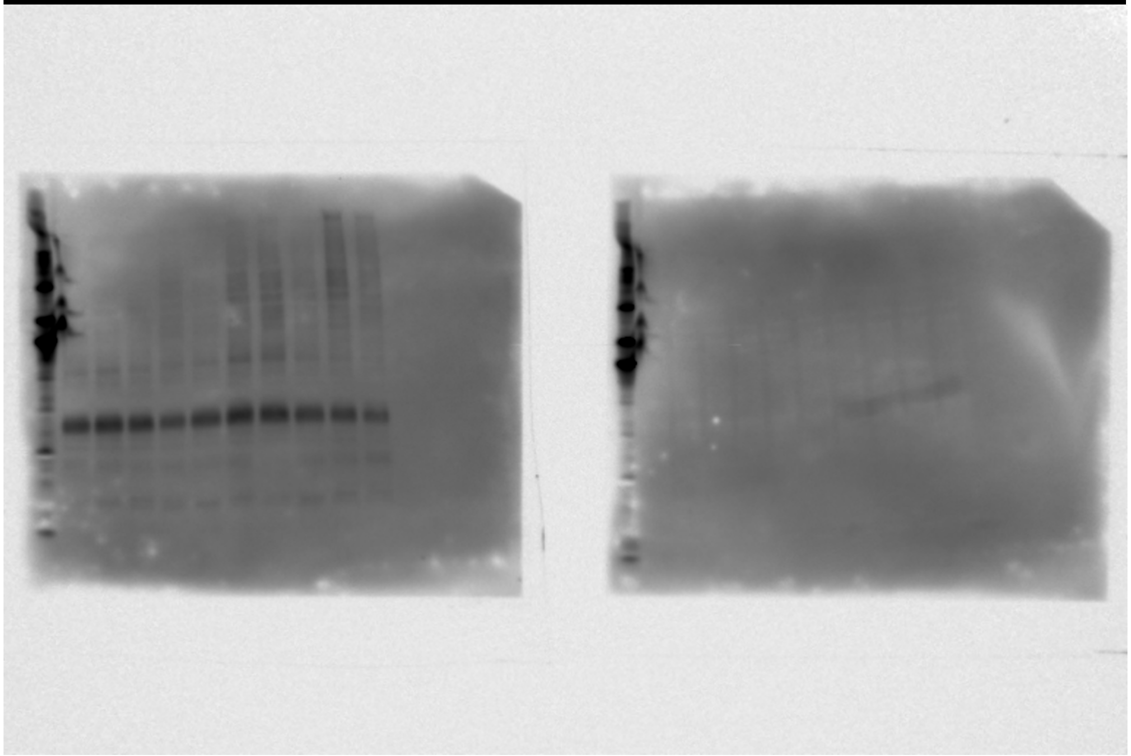

Supplement: S1 Raw images — (PDF) [file pone.0253184.s003.pdf]
